# Surface-Enhanced Raman Spectroscopy (SERS) Based Biological and Environmental 2D and 3D Imaging

**DOI:** 10.1021/acsenvironau.4c00149

**Published:** 2025-05-30

**Authors:** Qishen Huang, Huiyuan Guo, Wei Wang, Seju Kang, Peter J. Vikesland

**Affiliations:** † School of Chemistry and Chemical Engineering, Beijing Institute of Technology, Beijing 100081, China; ‡ Department of Chemistry, State University of New York at Binghamton, Binghamton, New York 13902, United States; § School of Civil and Environmental Engineering, Georgia Institute of Technology, Atlanta, Georgia 30332, United States; ∥ Eawag, Swiss Federal Institute of Aquatic Science and Technology, Dübendorf CH-8600, Switzerland; ⊥ Civil and Environmental Engineering and Institute of Critical Technology and Applied Science (ICTAS) Sustainable Nanotechnology, Virginia Tech, Blacksburg, Virginia 24061, United States

**Keywords:** SERS, imaging, nanoprobes, nanostructures, biological sensing, environmental sensing, microenvironments, analytical methods

## Abstract

Surface-enhanced Raman spectroscopy (SERS) imaging is a highly sensitive, spatially resolved tool for biological and environmental analysis. SERS imaging combines molecular fingerprinting with real-time, in situ detection, with the capacity to address key questions around analyte identification, concentration, and distribution. In biological systems, SERS imaging has enabled sensitive detection of nucleic acids, proteins, and biomarkers. Notable progress includes the detection of miRNAs through nanoassembly and disassembly techniques, as well as bioorthogonal chemistry and antibody-conjugated methods for protein and enzyme imaging. These approaches, along with integration of complementary imaging techniques, have improved SERS imaging for in vivo studies in plant and animal cells. Additionally, SERS imaging of pathogens reveals their distribution and behavior in cellular environments. For environmental applications, SERS imaging has been used to track pesticides, nanoparticles, and heavy metal ions, providing critical insights into contaminant transport and transformation. Furthermore, SERS-based pH and reactive oxygen species (ROS) imaging delivers spatially resolved data on reactive species in biological and environmental microenvironments, aiding in understanding their dynamic roles in various processes. Despite its advantages, SERS imaging faces several challenges. By addressing its limitations, SERS imaging holds promise for broad application in contaminant monitoring, clinical diagnostics, and real-time biological analysis.

## Graphical abstract

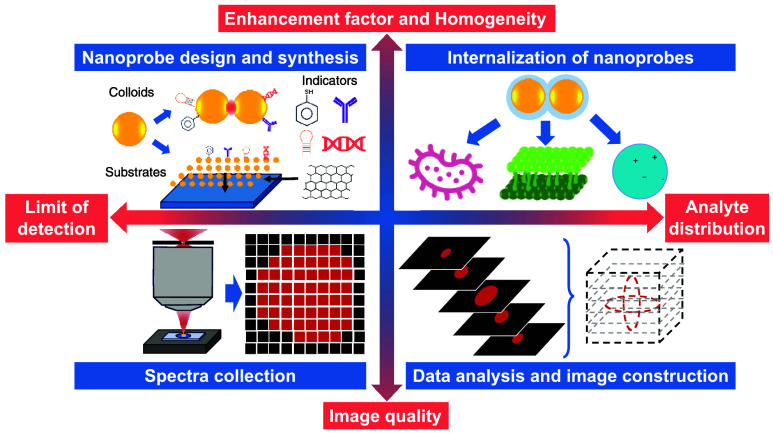

## 1 Introduction

Raman spectroscopy, discovered by C.V. Raman in 1928, has become an essential tool in the biological and environmental analytical chemistry due its ability to provide rich molecular fingerprint information with exceptional sensitivity.^1^ As an advancement of Raman spectroscopy, surface-enhanced Raman spectroscopy (SERS) imaging leverages nanostructured substrates or colloidal nanoparticles to enhance Raman signals, enabling spatially resolved, highly sensitive detection of target analytes.^2^ Initially developed for biological applications, SERS imaging has advanced in fields such as biomolecular detection, in vivo cellular analysis, and pathogen tracking. Inspired by these advancements, SERS imaging has expanded into environmental research,^2^ where it faces unique challenges, including contaminant identification in complex matrices, tracking the spatial and temporal evolution of pollutants, and adapting to ever-changing environmental conditions, which limit replicability in complex open systems such as actual environmental scenarios. Despite these differences, the underlying principles remain interconnected, both biological and environmental SERS imaging rely on highly sensitive, nondestructive detection methods to visualize molecular interactions, making SERS imaging a powerful technique for investigating complex systems across disciplines.

Biological and environmental analytical methods typically attempt to address one of four fundamental questions: (1) What analytes are present in a given sample? (2) What are their concentrations? (3) What are their spatial distributions? and (4) How do they change over time? Many techniques have been developed for biological or environmental analysis; however, the majority of these techniques only answer one or two of these questions at a time. For example, chromatography methods, such as gas chromatography (GC) and liquid chromatography (LC), are widely used to identify analytes and determine their concentrations by separating complex mixtures; however, these methods cannot readily provide spatial information about analyte distributions within a sample. Fluorescence spectroscopy is often used to detect molecules and measure their concentrations based upon their intrinsic luminescence. However, many environmental contaminants are not luminescent and many luminescent molecules are not necessarily present in biological and environmental samples, and the use of external fluorophores may cause photobleaching and chemical degradation.^3^ Accordingly, the molecular information provided by fluorescence spectroscopy is often limited.^4^ Similar challenges beset other spatially resolved techniques such as magnetic resonance imaging (MRI), computed tomography (CT), and ultrasound.

Raman spectroscopy has the potential to address all four of these questions simultaneously. However, the universal application of traditional Raman spectroscopy is limited by its low sensitivity and potentially interferent high fluorescence backgrounds.^5^ Through the advancement of nanotechnology and nanomaterials, the coupling of Raman spectroscopy and nanotechnology gave rise to surface-enhanced Raman spectroscopy (SERS)—50 years old as of 2024. This hybrid approach retains the advantages of Raman spectroscopy and overcomes many of its limitations thanks to the enhanced Raman scattering that occurs for target molecules in the vicinity of nanostructures and nanostructured (i.e., roughened) materials. SERS enhancement arises due to electromagnetic and chemical effects.^6,7^ Electromagnetic enhancement occurs when noble metal surfaces (e.g., Ag, Au, and Cu) are in close proximity to an analyte and amplify the electromagnetic field by orders of magnitude. Electromagnetic enhancement is highly dependent on interparticle distance, which can often be optimized to achieve high Raman enhancement. Chemical enhancement relies on charge transfer between a nanosubstrate and the target analyte. In addition to these well-established mechanisms, additional signal enhancement mechanisms have been proposed and are currently under investigation.^8,9^ The overall signal enhancement of SERS relative to normal Raman spectroscopy is defined by the enhancement factor (EF): $\mathrm{EF}=\frac{I_{\mathrm{SERS}}/N_{\mathrm{SERS}}}{I_{\mathrm{Raman}}/N_{\mathrm{Raman}}}$ . Here, *I*_SERS_ and *I*_Raman_ denote the measured signal intensity in the SERS vs normal Raman sampling volumes, and *N*_SERS_ and *N*_Raman_ reflect the analyte concentration within the sampling volume.^10^

Coupled with confocal Raman microscopy, SERS imaging is one of the few analytical techniques that can answer all four questions demanded by biological and environmental analysis. The confocal pinhole along with microscope objectives of various magnitudes and numerical apertures (NA) determines the spatial resolution of the confocal point. Switching between different lasers (typically 532, 633, and 785 nm) provides optimization of SERS signals relative to the ever-present fluorescent background. In addition, the grating of the spectrometer determines the spectral range and the spectral resolution. Optimization of optical parameters, along with sample preparation, laser power, and integration time are key for successful imaging. On top of these, SERS is able to detect and discriminate analytes from complex unknown systems due to the high sensitivity and high signal-to-noise ratios of the molecular fingerprints of individual molecules.^11^ Further, SERS can track molecular reactions, with minimal sample preparation and high temporal resolution, due to its capability to detect reactants, intermediates, and products within short data collection times.^12^ SERS can map molecular distributions in two-dimensional (2D) and three-dimensional (3D), and thus can serve as a platform for chemical imaging within micron and submicron scale environments (e.g., single living cells,^13,14^ individual microdroplets^15–20^ ). Properly functionalized SERS nanoprobes can be readily encapsulated into other materials (e.g., silica, poly­(ethylene glycol) (PEG), bovine serum albumin (BSA)^21,22^ ) to enable both high photostability and biocompatibility such that SERS probes are suitable for noninvasive and precise biomolecule tracking within live cells and environmental samples. SERS can also be utilized for real-time imaging, through which molecular dynamics, such as molecular movement and molecular transformations over time, can be observed at high spatial resolution, even at the single molecule level.^23–27^ Overall, SERS imaging provides sufficient resolution to monitor microenvironments and track molecular distributions.

The aim of this review is to provide a critical discussion of the current state of the field of SERS imaging. We start with the workflow for SERS imaging and then discuss the current research status of SERS imaging for biological and environmental analysis. Given the chronological development and maturity of the field, we begin with SERS imaging of biological analytes in biological matrices, followed by the imaging of reactive species, which have recently gained interest in both biological and environmental systems, and then move on to SERS imaging of environmental contaminants for environmental analysis. We identify limitations and challenges of previous studies, and propose potential solutions to guide future research. Furthermore, we provide new perspectives on knowledge and analytical gaps that need to be filled. Compared to extensive applications of SERS imaging for biological analysis, fewer publications can be found in the environmental realm. The progress of SERS applications in environmental imaging has lagged behind while tremendous advances have been made in biological imaging over decades. Accordingly, this review article emphasizes the potential translation of SERS imaging techniques developed for biological systems to those for environmental systems so that the full potential of SERS imaging for environmental analysis can be reached.

## 2 Typical Workflow for SERS Imaging

The workflow for SERS imaging typically includes four steps: (1) design and synthesis of the imaging nanoprobe/substrate; (2) introduction of the nanoprobe/substrate into the imaging system; (3) instrument setup and image/spectrum collection; and (4) data analysis and map construction (Figure 1).

**Figure 1:**
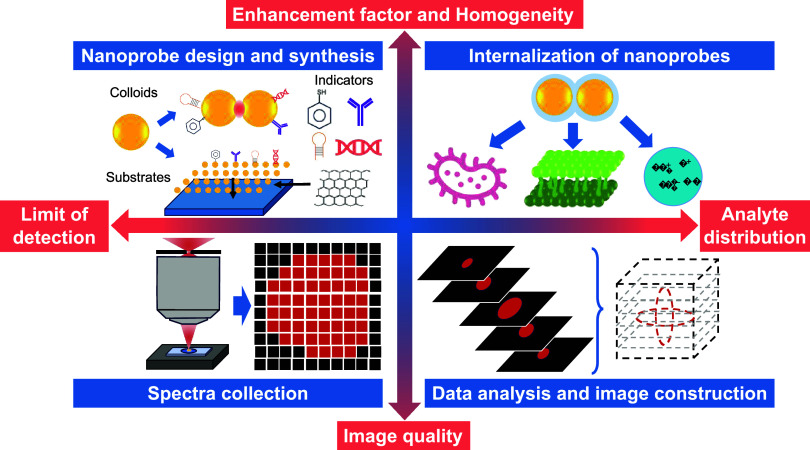
Schematic of the typical workflow for SERS 2D and 3D imaging in biological and environmental analysis.

### 2.1 Design and Synthesis of Imaging Nanoprobes

Herein, we define a nanoprobe (i.e., nanosensor) as a composite nanostructure that is either indicator-free (i.e., label-free) or indicator-functionalized (i.e., labeled). Depending on the desired application, nanostructures that are available for SERS imaging include: colloidal nanoparticles (including nanospheres,^14,15^ nanostars,^28^ nanorods,^29^ or core–shell structures^30^), nanoprobes immobilized on a surface,^31^ nanoprobes produced in situ,^32^ and microneedles.^33^ Commonly used base materials that provide high EF include gold,^30^ silver,^34^ gold/silver hybrids,^35^ as well as other elements (e.g., silica).^30^

If the goal of SERS detection is to detect the nonspecific intrinsic signals arising from molecules in the imaging system, then indicator-free nanoprobes should be used for direct SERS imaging. In many cases, however, target molecules exhibit minimal or no Raman signals and thus indicator-assisted SERS imaging is required for target-specific detection. We note that, in some articles, the term “Raman reporter” instead of “indicator” is used. The two terms are generally interchangeable across most scenarios. These terms reflect molecules whose Raman signals provide a desired capability. Herein, we will use the term “indicators” to represent such molecules. The labeled indicator can be multifunctional, to provide high sensitivity and/or molecular specificity if the analyte interacts with the indicator specifically and modifies its SERS signal. Three designs are typical: (1) indicator for signal detection; (2) molecules for target recognition and specific detection; and (3) stabilizing agents to increase nanoprobe stability and biocompatibility.

Regardless of which nanoprobe is chosen for SERS imaging it must generally meet three requirements.

#### 2.1.1 Capacity to Generate Sufficient SERS Hotspots

SERS hotspots form when the frequency of the excitation laser couples with (i.e., overlaps with) the localized surface plasmon resonance (LSPR) frequency of the nanoprobes and generates an enhanced Raman signal of an analyte.^6,7^ Engineering of sufficient numbers of SERS hotspots is required to ensure high quality SERS imaging. SERS hotspots can be created various ways. Two basic principles are that (a) nanoparticles within close vicinity of one another exhibit higher SERS enhancements than single isolated nanoparticles; and (b) EF increases with a decrease in interparticle distance.^36^ Accordingly, nanoparticle aggregation, achieved by introduction of aggregating agents, is a commonly used strategy to generate hotspots. External agents and the target analyte itself can be used as aggregating agents. Target-induced “off/on” aggregation not only creates hotspots, but also has been used for SERS detection due to the significant increase in signal intensity.^37,38^ Aggregation is a stochastic process that is difficult to reproducibly control and thus many aggregation assays are susceptible to signal inhomogeneity. Nanoparticles can also be fabricated onto surfaces or tips and consequently the interparticle gap can be precisely controlled. These types of nanosensors are usually fabricated via top-down lithographic techniques, such as e-beam lithography, photolithography, nanoimprint lithography, or 3D printing.^39–41^ Further details and examples of SERS hot spots can be found in another recent review.^42^

#### 2.1.2 Even Spatial Distribution of Nanoprobes

Homogeneously distributed nanoprobes ensure that SERS imaging describes the analyte distribution instead of being biased by the spatial heterogeneity of the nanoprobes. This requirement is critical for improving quantitative nanoprobe performance (i.e., low variation and high reproducibility). It is relatively simple to achieve an even distribution of nanoprobes fixed to a surface, but it is inherently more challenging for colloid-based nanoprobe suspensions. Nanogap-enhanced SERS probes are designed to achieve controlled and uniform enhancements using colloids. Various linker molecules, such as DNA, block copolymers, etc., as well as different morphologies, including nanoparticle packing, nanodumbbells, and core–shell structures, have been developed to achieve uniform nanogap enhancements.^43,44^ The recently developed hot-spot normalization method normalizes the SERS signal from the indicator/analyte to that of the elastic scattering peak, which is cutoff by the notch filter at low wavenumbers. Since both the elastic peak and the SERS signal share the same enhancement effect from the hot-spot, the hot-spot normalization method can potentially correct biases resulting from heterogeneous nanoprobe distributions.^45,46^ Nonetheless, the production of spatially uniform colloidal nanoprobes needs to be addressed in the future.

#### 2.1.3 Close Contact with Analytes in the Imaging Environment

The EF decreases by orders of magnitude a few nanometers beyond the hotspot and thus it is crucial to control the distance between nanoprobes and analytes either via direct covalent bonding or cross-linking. In addition, the intended imaging environment plays a decisive role in the design and synthesis of nanoprobes: colloidal probes that can enter cells may not work well when mapping extracellular components or cellular activities due to the possible signals arising from the intracellular area. Similarly, nanoprobes fixed on a substrate are not suitable for intracellular imaging. Therefore, to design effective nanoprobes, factors such as the imaging environment and the location of the analytes need to be considered a priori.

### 2.2 Introduction of Nanoprobes into the Imaging System

The efficacy of nanoprobes for SERS imaging is not only dependent on the design and synthesis of the nanoprobes, but also on their efficient introduction into the imaging system. The ideal imaging condition is that the nanoprobe concentration is sufficient to evenly cover all targets. Taking intracellular imaging as an example, nanoprobe internalization and target recognition by the nanoprobe are important questions to address. These needs should be considered prior to the design and synthesis of nanoprobes. For instance, prior work has utilized cell-penetrating peptides to functionalize pH nanoprobes and facilitate their entrance into cells.^14^ Further, organelle-targeting peptides have been used to modify nanoprobes to achieve pH imaging in specific organelles (e.g., nucleus, mitochondria, and lysosome).^47^ In the absence of cell-targeting peptides and organelle-targeting peptides, nanoprobes often exhibit low internalization efficiency and end up primarily found within endosomes.^48^ Another knowledge gap is that few studies to date have quantified the percentage of analytes detected by nanoprobes, which is arguably due to lack of standards and the complexity of the different imaging environments. Accordingly, there is high potential that the accessibility of nanoprobes to analytes often influences imaging. To address this bottleneck, further investigation and improvement are required.

### 2.3 Instrument Setup and Image/Spectrum Collection

Due to its high spatial resolution and sensitivity, benchtop Raman spectroscopy coupled with a confocal microscope is the typical setup providing the capacity for 2D and 3D imaging.^49^ Nonconfocal Raman spectroscopy is potentially able to perform 2D scans. To achieve the best performance with confocal Raman spectroscopy, parameters including laser wavelength, laser power, integration time, microscope objective lens, and spatial resolution should be optimized. Moreover, machine-learning assisted methods have been recently developed for SERS imaging for improved efficiency and capability for classification.^50^ Interested readers can refer to a published review on how to use experimental and computational approaches to optimize SERS setup and parameters.^51^

### 2.4 Data Analysis and Image Construction

Following image collection, the next question is how to analyze the data and use it to create SERS images that reflect reality as closely as possible. In indicator-assisted SERS, the method to convert the indicator signal into analyte concentration is a key question. In indicator-free SERS, the spectral peaks originate from the analytes directly; however, signal intensity does not necessarily correlate with analyte concentration because the SERS hotspot distribution can affect the signal intensity. To improve the quantitative capacity of SERS imaging, internal standards are commonly required. Peaks independent of the analytes, but dependent on the hotspots can be used for calibration. Previous studies have shown that a low wavenumber peak reflecting elastic scattering can be used as an internal standard to reduce the variation caused by spatial hotspot heterogeneity.^45,52,53^ Data analysis also influences the quality of the constructed maps. SERS map resolution is dependent on the spatial resolution of the instrument; however, the map resolution can be improved via data analysis. For example, Ravindranath et al. used a 100× objective with spatial resolution of ∼1 μm^2^.^25^ They scanned a 6 × 6 μm^2^ area with a 40 × 40 grid and a step size of 125 nm. In many cases, the data sets acquired via SERS imaging are large and complex, which requires effective ways to extract the desired information. Historically, one of the most apparent peaks for a given analyte has been selected to build SERS images. However, molecules often have multiple characteristic peaks and whether the selected peak is the most stable and representative needs to be considered. So far, only a limited number of papers have provided justification of their peak selection for imaging. Incorporating data processing tools, such as machine learning approaches into SERS imaging analysis may help achieve full use of the rich molecular information stored within SERS spectra. Thrift and Ragan applied a convolutional neural network (CNN) model to perform concentration regression on bundles of SERS maps in the single-molecule detection regime, achieving a limit of quantification down to 10 fM for Rhodamine 800. These results pave the way for unambiguous analysis of large spectral data sets and the use of SERS in ultralow concentration chemical detection.^54^ A thorough and insightful review was published recently on the applications of machine learning and artificial intelligence for Raman spectral analysis.^55^

## 3 Applications of SERS Imaging in Biological Sensing

SERS imaging of a variety of biological analytes including nucleic acids, proteins, enzymes, pathogens, and living cells has attracted great interest across the medical, analytical, agricultural, pharmaceutical, and environmental sectors. The bioactivity of pathogens and cells can often be explained by detection of associated biomolecules. High-resolution images of biomarkers associated with a specific disease, or precise images of tumors are key for diagnostic decision making and image-guided therapy, which is highly important as a diagnostic tool for biomedical applications.^56^

### 3.1 SERS Imaging of Nucleic Acids

Nucleic acids, DNA and RNA, are polymers of nucleotides. Studies have shown that adenine, a nucleobase, plays an important role in the SERS spectra of gene sequences,^57,58^ and is capable of identifying DNA or RNA fragments.^59–61^ By imaging the SERS map according to the distinct vibrational signatures of two isotopologues of adenine (^14^NA and ^15^NA), Zong et al. tracked nucleotide degradation due to starvation stress.^62^ They found that adenine was most concentrated in the pericellular region near the outer cell wall under starvation conditions.

Micro RNAs (miRNAs), whose discovery was honored with the 2024 Nobel Prize in Physiology or Medicine, are crucial short nucleic acid sequences involved in biological processes.^63,64^ For example, RNA interference (RNAi), where micro (miRNA) and small interfering RNA decrease mRNA (mRNA) activity by preventing translation.^63^ SERS imaging methods based on nanoassembly between nanoprobes and target miRNA have been developed to detect the presence of miRNA. Zhou et al. utilized 4-mercaptobenzonitrile and 4-ethynylbenzene-thiol modified gold nanoparticles as SERS probes for real-time multiplexed imaging of intracellular miRNA (Figure 2A).^64^ Specific binding between locked nucleic acid (LNA) sequences on the SERS probes and target miRNA molecules formed dimeric nanostructures, significantly enhancing Raman scattering and enabling high-resolution imaging of multiple miRNA distribution within cells. Crawford et al. developed an inverse molecular sentinel (iMS) nanoprobe for in vivo SERS imaging of miRNA in plants.^65^ The 5′ end of the iMS sequence was modified on the surface of nanoparticles and the 3′ end was labeled with Cy7 dye. Upon binding with target miR156, the iMS sequence formed a hairpin (i.e., single strand DNA with stem-loop structure) conformation, bringing the Cy7 functionalized end into close proximity with the nanoparticle surface thus significantly enhancing the SERS signal. Lu et al. reported a single plasmonic microbead functionalized with the S9.6 antibody and DNA probe for universal and specific binding DNA/miRNA for multiplex miRNA analysis.^66^ Sub-pM target miRNA on this single microbead was successfully imaged with multiplexed capability.

**Figure 2:**
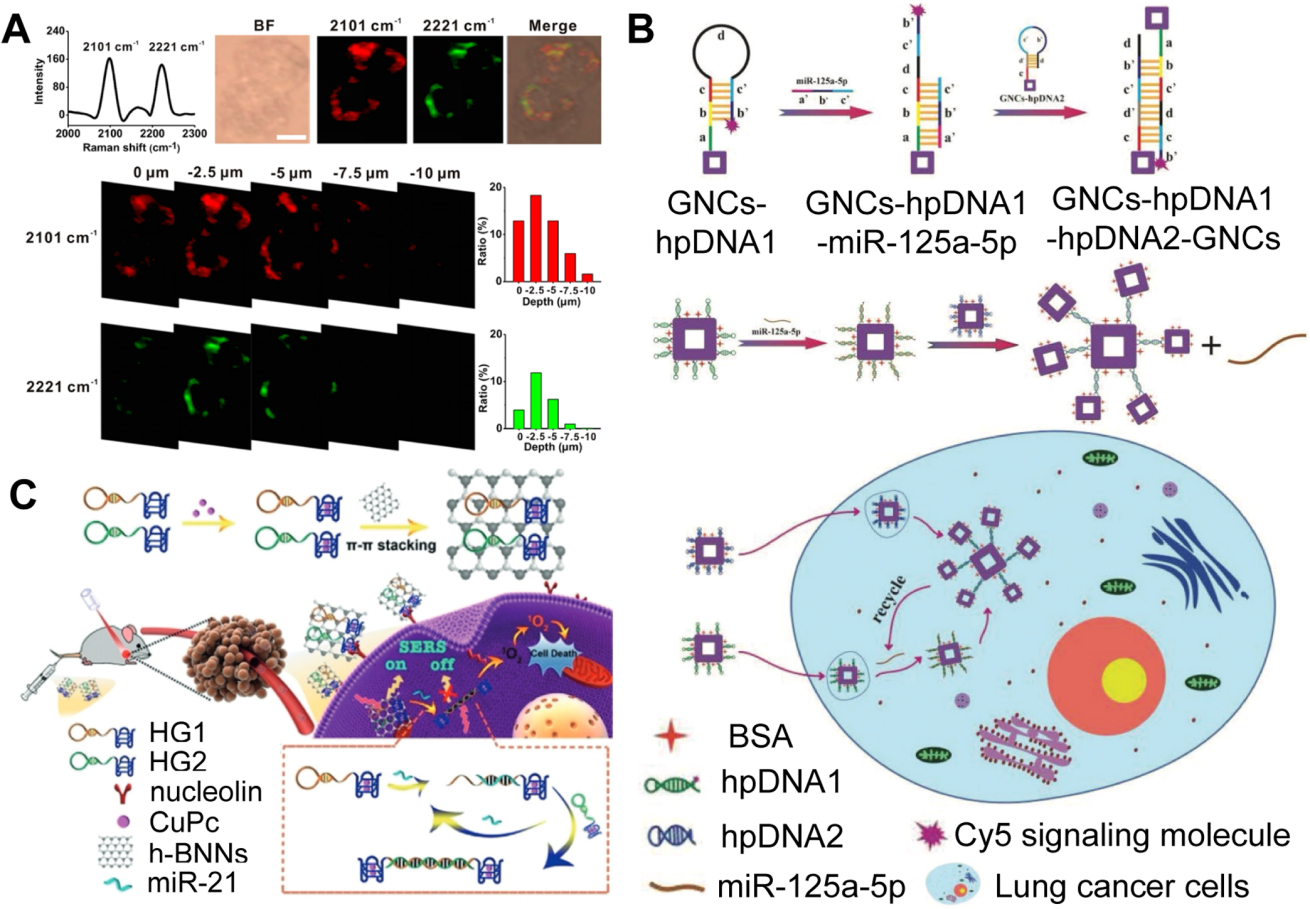
(A) Multiplexed image of miR-21 (2101 cm^–1^) and miR-155 (2221 cm^–1^) in living cells (Reproduced from ref 63. Copyright 2017 American Chemical Society). (B) Schematic of the signal amplification SERS assay with catalytic hairpin assembly (CHA) reaction and the application of hairpin DNA (hpDNA) functionalized gold nanocages (hpDNA-functionalized-GNC) for miRNA (miR125a-5p) SERS imaging in lung cancer cells (Reproduced with permission from ref 67. Copyright 2019 Royal Society of Chemistry). (C) Illustration of the synthesis of copper phthalocyanine (CuPc)—hexagonal boron nitride nanosheet (h-BNNS) substrate and the mechanism of miR-21 SERS imaging in living cells (Reproduced with permission from ref 70. Copyright 2019 John Wiley and Sons).

Building on catalytic hairpin assembly (CHA), researchers have developed various SERS probes for improved nucleic acid resolution. Wang et al. developed gold nanocages (GNCs) induced by CHA using single strand hairpin DNA (hpDNA1 and hpDNA2).^67^ The hollow interiors and perforated walls of the GNC provided ample space for Raman-sensitive molecules, significantly enhancing SERS sensitivity. These GNCs have excellent biocompatibility and can protect the DNA structure bound to the surface. Using Cy5 as a Raman reporter, the hpDNA1-hpDNA2-GNC nanoprobes enabled SERS imaging of miR-125a-5p in both a normal lung epithelial cell line (BEAS-2B cells) and a lung cancer cell line (A549 cells) (Figure 2B).^67^ In another study, a similar structure was used to achieve long-term monitoring of intracellular miRNA.^68^ Liu et al. developed target miRNA-triggered CHA-induced AuNP-Au nanodumbbell core–satellites, which allow the detection of low-abundance miRNAs in living cells, revealing higher expression of mir-1246 in HeLa cells compared to MCF-7 cells.^69^ In the presence of target miRNA, hybridization between hairpin 1 (H1) on the AuNPs and hairpin 2 (H2) on the Au nanodumbbells can form a stable H1–H2 duplex and generate new hot spots that provide intensely enhanced SERS signals.

The disassembly of nanomaterials can also enable high-resolution SERS imaging. Liu et al. created a multifunctional integrated nanoplatform with a hexagonal boron nitride nanosheet (h-BNN) substrate and copper phthalocyanine (CuPc) as the Raman indicator for SERS imaging of miR-21 (Figure 2C).^70^ The presence of miR-21 breaks the interaction between CuPc and h-BNNS leading to a decreased CuPc signal, thereby allowing quantification and imaging of miR-21 in cell. Using a similar method, Li et al. developed arrowhead nanorod (NR) dimers, functionalized with two single-stranded DNA probes (DNA01 and DNA02) and a Raman indicator (4-aminothiophenol, 4-ATP) for in situ SERS imaging of miRNA in living cells.^71^ The reduction in SERS intensity due to dimer disassembly was applied in SERS mapping to accurately quantify and locate intracellular miR-21 and miR-203b.

SERS can be combined with other techniques to obtain rapid and direct visualization of intracellular miRNA. Ye et al. designed dual-signal switchable (DSS) nanoprobes using a fluorescence-Raman signal switch.^72,73^ In the absence of miRNA, the probe was in a state of fluorescence signal-on and SERS signal-off. In the presence of target miRNA molecules, the probe was switched to the state of fluorescence signal-off and SERS signal-on. Mapping of the fluorescence signal was used to show cellular uptake and distribution of the probes whereas the maps based on the SERS signal were used to show the distribution of target miRNA molecules. This design can be utilized to track the intracellular behavior of the probe and quantitatively determine miRNAs in situ.

### 3.2 SERS Imaging of Proteins, Enzymes, and Small Biomolecules

Certain proteins, enzymes, and small biomolecules (smaller than nucleic acids) are often recognized as biomarkers of normal biological, pathogenic, and pharmacological processes. In SERS imaging of bioanalytes, interference-free imaging strategies are desired. Most Raman vibrational modes from such endogenous biological molecules are found in the mid- to low-wavenumber region (<1800 cm^–1^). Accordingly, Raman reporters with intense Raman signals in the high-wavenumber region (>2000 cm^–1^) have been designed and applied for noninvasive SERS imaging of living cells.^74^

Following the principle of bioorthogonal chemistry (i.e., chemical reactions occur inside living cells without interference from biomolecules), alkyl-modulated Raman reporters were designed for live cell imaging,^74^ since alkynes exhibit a distinct and strong Raman signal in the silent region of 1800–2800 cm^–1^ where most cellular components do not show Raman signals (Figure 3A).^75^ Using 4-mercaptophenylboronic acid (MPBA) modified silver nanoparticles (AgNPs), Cong et al. distinguished higher levels of sialic acid expression in cancer cell lines via the detection of sialic acid due to the specific binding between MPBA and sialic acid.^76^ Using various bioorthogonal Raman indicators such as azides, alkynes, and carbon-deuterium (C–D) bonds, Wang et al. successfully obtained SERS images of cell surface proteins, glycans, and lipids using arrays of AuNPs as SERS-active substrates.^77^ In this work, the Raman indicator, instead of functionalization on nanoparticles, was added to the culture media and was internalized by cells through their metabolic activity.

**Figure 3:**
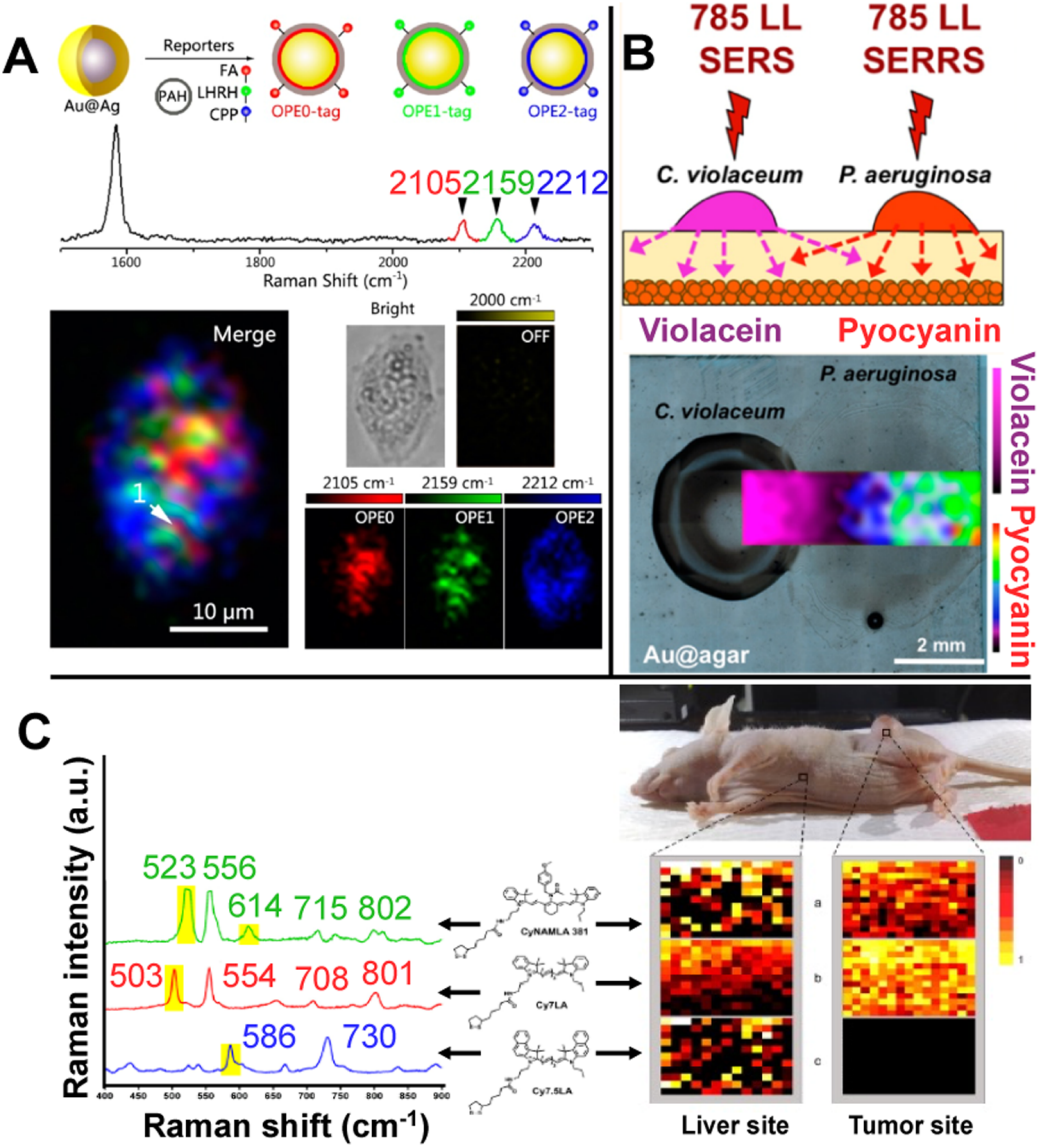
(A) Three-color SERS imaging in live HeLa cells using SERS probes from the alkyne SERS palette, achieved through PAHylation and peptide/small-molecule modifications of Raman dye-coded Au@Ag-NPs (dyes: OPE0 (red), OPE1 (green), and OPE2 (blue)) (Reproduced from ref 75. Copyright 2016 American Chemical Society). (B) Schematic illustration of in vivo surface-enhanced resonance Raman scattering (SERRS) spectroscopy multiplexed detection of violacein and pyocyanin in CV026 and PA14 bacterial cocultures grown on Au@agar. Visualization of violacein and pyocyanin was carried out following illumination of the bacterial colonies on the plasmonic sensor with a NIR 785 nm laser (Reproduced from ref 94. Copyright 2017 American Chemical Society). (C) Normalized SERS spectra of CyNAMLA-381, Cy7LA and Cy7.5LA after chemisorption on AuNPs (left) and multiplex SERS mapping images generated corresponding to the labeled peak of the three indicators (right) (Reproduced from ref 100. Copyright 2012 Elsevier).

The formation of nanoparticle dimers induced by target biomolecules is another strategy to image biomolecules by SERS. Wissler et al. synthesized SERS-inactive AuNP monomers modified with Raman indicators.^78^ In the presence of the GST-Survivin-His6 protein, AuNP dimer formation is induced thus activating SERS response due to plasmonic coupling between AuNPs. Ardini et al. linked colloidal silver nanoparticles with alkyne-dopamine adducts to induce formation of clusters with high electric field enhancement and strong SERS signals for alkynes and dopamines.^79^ The strong alkyne signal in the cell silent region distinguished dopamine across the cytoplasm with high spatial resolution. Membrane protein (i.e., Met and TβRII) dimerization on living cells was imaged using SERS tags and AuNPs-based dual-recognition probes and SERS tags.^80^ The dimerization of proteins resulted in the proximity ligation-assisted CHA-based networking of probes and SERS tags to form nanostructures with significantly enhanced SERS effect.

SERS imaging based on antibody-conjugated immunoassays is promising to measure biomolecule distribution and internalization in cells,^81,82^ and has been widely used to detect a variety of biomolecules.^83–85^ By modifying GNRs with 4-mercaptobenzoic acid (4-MBA) and an antibody, Xiao et al. revealed the spatial distribution and the dynamic change in epidermal growth factor receptor (EGFR) levels in living breast cancer cells.^86^ They detected an overexpressed receptor, tyrosine kinase, in human cancer cells. Using a similar method, SERS imaging of EGFR and human epidermal growth factor 2 (HER2) on the lumenal surface of the rat esophagus was obtained.^87^ The distribution of the tumor-associated surface biomarker (CD44) was characterized on the surface of HCT116 cells using hyaluronic acid functionalized high narrow nanogap-containing Au@Au core–shell SERS tags.^88^ Furthermore, imprinted polymer layers on the SERS probe can act as an artificial antibody and specifically bind with biomolecules in the cells.^89^

SERS probes can also be designed to detect biomolecules through cleavage, typically by linking Raman indicators and nanostructures through aptamers or peptides. In the presence of target biomolecules, the linkage can be cleared, and Raman indicators are released from the SERS probe reducing its SERS intensity. Si et al. developed a ratiometric SERS nanosensor, Au/Ag nanoparticles modified with 3-[4-(Phenylethynyl)­benzylthio] propanoic acid (PEB) using a ssDNA linkage, to probe endonuclease activity under in vitro and living cell conditions.^90^ In this study, overexpression of endonuclease during cell apoptosis was identified via a decreased SERS signal. Through a similar approach, substituting ssDNA with a peptide as the linker, SERS imaging of caspase 3 in live cells and tissues was obtained.^91^ Guo et al. designed the tetrahedron probe to detect telomerase (TE) and epithelial cell-adhesion molecule (EpCAM) simultaneously in living cells and differentiated the cancer cell lines HeLa, MCF-7, and normal primary uterine fibroblast cells.^92^ Assembled by DNA frames, the tetrahedron probe consisted of three 15 nm AuNPs and two 30 nm AuNPs. Raman indicators, 4-nitrothiophenol (NTP) and 4-methoxybenzyl mercaptan (MATT) linked with a TE or EpCAM aptamer respectively, were functionalized onto two 30 nm AuNPs. Fifteen nm AuNPs modified with 4-ATP was regarded as an internal reference and located in the middle of the tetrahedron probe. In the presence of TE or EpCAM, the designated building blocks disassembled, the release of the 30 nm AuNPs led to a decrease in the SERS intensity of NTP or MATT.

Label-free SERS imaging is also applicable for characterizing the intracellular distribution of biomolecules,^93^ and investigating metabolic interactions between microbial populations.^94–96^ Li et al. used label-free SERS imaging and revealed the distribution of CD36 protein on the plasma membrane and in lung tumor-bearing mice. CD36 protein–ligand (KOdiA-PC) was used to recognize a transmembrane receptor (CD36) that is greatly repressed in carcinoma-associated fibroblasts (CAFs).^93^ Using Au@agar substrate, Bodelón et al. detected violacein and pyocyanin produced by bacterial colonies of (, CV026) and (, PA14), respectively (Figure 3B).^94^ They also observed the production and spatial distribution of indole and pyocyanin in cocultured populations of and .^95^ For extracellular metabolites, Lussier reported a plasmonic nanosensor decorated with gold nanoraspberries that can be placed near the vicinity of the living cells at a distance of 30 μm. Dynamic SERS was monitored continuously on a single region near the tip of the nanosensor and achieved pyruvate, lactate, ATP, and urea identification with low interference in a complex and biologically relevant medium.^97^

### 3.3 In Vivo SERS Imaging of Animal and Plant Cells

Delineation of living cells through the collection of accurate images is of clinical significance for preoperative and intraoperative surgical processes. Generally, live cell imaging is conducted in one of two ways: in vitro or in vivo. For in vitro studies, the target analytes are isolated from their biological context and further experiments are conducted in a test tube. Conversely, for in vivo studies, the biological entities are tested inside a cell without extraction.

Raman indicators active in the near-infrared (NIR) region have been discovered for in vivo SERS imaging within cells. NIR excitation lasers (e.g., 785 nm) enable noninvasive detection and deep penetration into cells or human tissues.^98^ The Chang group recently synthesized a tricarbocyanine derivative (CyNAMLA-381) with large NIR absorption and good chemical stability, using it as a Raman indicator for in vivo cancer cell imaging.^99^ They obtained SERS images of HER2-positive CSKBR-3 cells with high sensitivity and specificity. The same group further conducted multiplex targeted in vivo cancer cell detection (Figure 3C) using three different tricarbocyanine derivatives (CyNAMLA-381, Cy7LA, Cy7.5LA).^100^ CyNAMLA-381- and Cy7LA-AuNPs were functionalized with antiepidermal growth factor receptor (anti-EGF) antibody for selective SERS imaging of oral squamous carcinoma cell (OSCC) and Cy7.5LA-AuNPs were conjugated with an anti-HER2 antibody as a negative control. Based on the characteristic peaks of CyNAMLA-381, Cy7LA, and Cy7.5LA (523, 503, and 586 cm^–1^), the images at the tumor sites showed high SERS intensities for anti-EGFR antibody conjugated CyNAMLA-381 and Cy7LA nanotags, but low intensities for anti-HER2 antibody conjugated Cy7.5LA nanotags. The images showed passive localization of all three nanotags at the liver site. To illustrate the potential of multiplexed SERS imaging for multiple living cells and biomarkers, Zavaleta et al. designed ten Raman indicator functionalized gold core and silica shell NPs was applied in living mice.^101^ All SERS spectra from the ten SERS nanoprobes were spectrally separated. Zeng et al. used AuNPs functionalized with two different alkyl-modulated Raman indicators (OPE1 and 4-MBN) and an aptamer against EGFR for multiplex SERS HeLa cellular imaging.^102^ Organometallic osmium carbonyl clusters (OM), Os3­(CO)­10­(μ-H)­2 were coupled with the AuNPs for SERS imaging of living cells.^103^ Based on the SERS images of 2030 cm^–1^ peak, they showed the specificity to the EGFR positive-OSCC, but not to EGFR negative-human ovary cancer cell line (SKOV-3).

For in vivo imaging, the photostability of the imaging agent during laser illumination is critical to ensure accurate live cell imaging. Therefore, silica encapsulated AuNPs have been used for delineation of tumor cells in the liver and spleen to increase the photostability of the SERS imaging agents.^104^ Silica coated AuNPs were conjugated with BPE as the Raman indicator, showing consistently high SERS signals for a long period. Such nanoprobes confer SERS imaging a huge advantage over fluorescence-based imaging technique due to the photobleaching of fluorophores (e.g., indocyanine green) upon laser illumination. With high photostability, nanoprobes can be used to identify cancerous lesions by contrast. With the same gold core and silica shell SERS nanoprobes functionalized with 44DP as the Raman indicator, a hand-held Raman scanner was applied to provide a SERS image of malignant brain tumors, in particular glioblastoma multiforme (GBM).^105^

SERS images of living cells can be improved though nanostructure design and incorporation of protective coatings. Gold nanobipyramid core and silver nanorod shell nanoparticles (AuNBP@AgNRs), modified with 4-MBA and FA, demonstrated excellent specificity of the SERS nanoprobes toward FA receptor-positive MGC-803 cells.^106^ Liu et al. developed folate-targeted hollow AuNPs (HAuNPs) as SERS nanoprobes to achieve clear SERS images for selective FA receptor-positive human nasopharyngeal epidermal carcinoma cell line (KB) and HeLa cells.^107^ Yang et al. investigated different ligand functionalized AuAg nanohollows for bladder normal (NIH/3T3 and SV-HUC1) and cancer RT4 (low-grade) and T24 (high-grade) cells.^108^ Using the CPBA or anti-EGFR/4-ATP/AuAg nanohollows, the SERS image of 1079 cm^–1^ from 4-ATP successfully identified the antigen distribution of T24 cells. To further increase the intensities of the Raman reporters, AuNPs with uniform intrananogaps (Au-NNPs) modified with DNA oligonucleotide spacer (e.g., subsequent 10 thymine, T_10_) were synthesized to achieve high-resolution/speed imaging for live HSC-3 cells using three different Au-NNPs probes targeting specific intracellular organelles (cytoplasm, mitochondria, and nucleus).^109^ Due to their high surface affinity to proteins, bare plasmonic NPs can develop a protein corona, which may alter the SERS spectra of living cells and impair image quality; however, applying a surface protective coating can minimize this nonspecific binding and improve SERS imaging quality. Sloan-Dennison et al. coated the surface of AuNSs with a small cyclic arginine-glycine-aspartic acid-phenylalanine-cysteine (RGDFC) peptide to prevent the formation of PC on the NP surface.^110^ This peptide provides a zwitterionic surface that hinders protein corona formation and enables specific detection of α_v_β_3_ integrin on fixed cells. The results show that the cyclic peptide protected against aggregation and amino acid adsorption to the AuNSs.

SERS imaging can be coupled with other imaging techniques to complement each limitation and provide additional information about living cells. The Gambhir group developed SERS and photoacoustic (PA) coupled imaging approaches for ovarian tumors using AuNRs. AuNRs show great promise as SERS and PA imaging agents. PA-based tumor imaging was used for presurgical tumor visualization and intraoperative SERS imaging allowed complete resection of tumor margins.^111^ They also synthesized triple modality MRI/PA/SERS imaging NPs for preoperative and intraoperative imaging of brain tumors.^112^ Brain tumor enhanced GFP-transfected human gliomablastoma cells (eGFP^+^U87MG) were visualized by the MRI/PA/SERS images using the synthesized NPs injected into the mice via the tail vein. For brain-tumor surgery, the MRI/SERS imaging of the tumor was developed using pH-responsive AuNPs.^113^ Cancer cell invasion acidifies the tumor extracellular fluid, causing polymer cleavage on AuNPs. This triggers AuNP aggregation via azide–alkyne click reactions in the acidic fluid, enabling highly sensitive MRI/SERS tumor imaging.

Compared to 2D imaging, 3D living cell imaging offers a more realistic view of cellular environments, enhancing our understanding of cellular complexity. Aberasturi et al. demonstrated this using Au nanostars (AuNSs) and nanorods (AuNRs) tuned to NIR absorption to achieve SERS imaging of dermal fibroblast (HDF) cells.^114^ The elongated HDF cells grew into skin-like 3D structures, actively internalizing and retaining positively charged SERS tags functionalized with poly-l-arginine hydrochloride. Imaging across the *z*-axis captured six layers of alternating SERS-labeled and nonlabeled cells, highlighting the power of 3D SERS imaging to reveal complex cellular arrangements. Similarly, Wang and Vikesland incorporated AuNPs to a hydrogel matrix to achieve a SERS-based hydrogel for 3D cell culture and imaging.^41^ The label-free SERS signal was collected in three dimensions and a random forest binary classifier was developed to discriminate Vero cell signals from the hydrogel background. Both 2D and 3D SERS images were obtained with a resolution of ∼3 μm and the maps obtained matched well with the cell optical images.

### 3.4 SERS Imaging of Pathogens

Pathogens—such as bacteria, viruses, protozoa, and fungi—are disease-causing organisms that can threaten human health when present in food or environmental settings. Public health demands have created an urgent need for rapid, sensitive pathogen detection techniques. SERS imaging offers significant advantages in this area, as it provides intrinsic fingerprint information directly related to the structure and spatial distribution of pathogens.

Label-free methods generate SERS images simply by mixing a SERS substrate with a pathogen, greatly enhancing the Raman signals of cellular components such as polysaccharides, amino acids, nucleic acids, lipids, and proteins. By mapping characteristic cellular component peaks, a clear image of a pathogen can be obtained that reflects morphology, component location, and concentration. Arnob et al. synthesized 3D SERS substrates for label-free imaging of and , achieving SERS maps that closely matched specific morphologies observed in microscopic images.^115^ By summing the spectral intensity of 50 SERS image pixels (each pixel contains the SERS spectrum from a 1.5 μm × 1.5 μm area that accommodates ∼50% of a bacterial cell), single-cell level detection limits were achieved. Huang et al. used AuNPs as surrogates for bacteriophage Phi6 to map its spatial distribution during microdroplet evaporation using SERS imaging. The study revealed a correlation between Phi6 infectivity and the coffee-ring effect during microdroplet evaporation based on the spatial distribution of AuNPs.^116^

To avoid interferences in label-free SERS imaging, biomolecule linkers that specifically attach nanostructures to a given pathogen have been developed. Fan et al. obtained SERS maps of *Salmonella* DT104 by modifying the surface of a nanoparticle with *Salmonella* DT104-specific antibody, M3038.^117^ Wang et al. used a trans-cyclooctene derivative of vancomycin, which binds Gram-positive bacteria by forming hydrogen bonds with the d-Ala-d-Ala motif of peptidoglycan on the bacterial cell wall, resulting in strong Raman signals from AuNP aggregation on the bacterial surface and suggesting potential for antimicrobial photothermal therapy.^118^ To further enhance the binding between SERS probes and pathogens, Zhou et al. developed an in situ synthesis method to produce AgNPs on the cell wall of bacteria to form Bacteria@AgNPs.^119^ This approach leveraged the negative charge of cell walls (due to teichoic acid in Gram-positive or lipopolysaccharides in Gram-negative bacteria) to adhere Ag^+^ ions, which were then reduced to AgNPs, increasing the Raman signal approximately 30-fold oversimple colloid suspensions. This spatially resolved SERS imaging of bacteria enabled precise SERS mapping of DSM 1116 in drinking water. Furthermore, such SERS images allowed discrimination between live Bacteria@AgNPs (showing strong SERS signals) and dead dead Bacteria@AgNPs (showing minimal signals, Figure 4A,B).^120^

**Figure 4:**
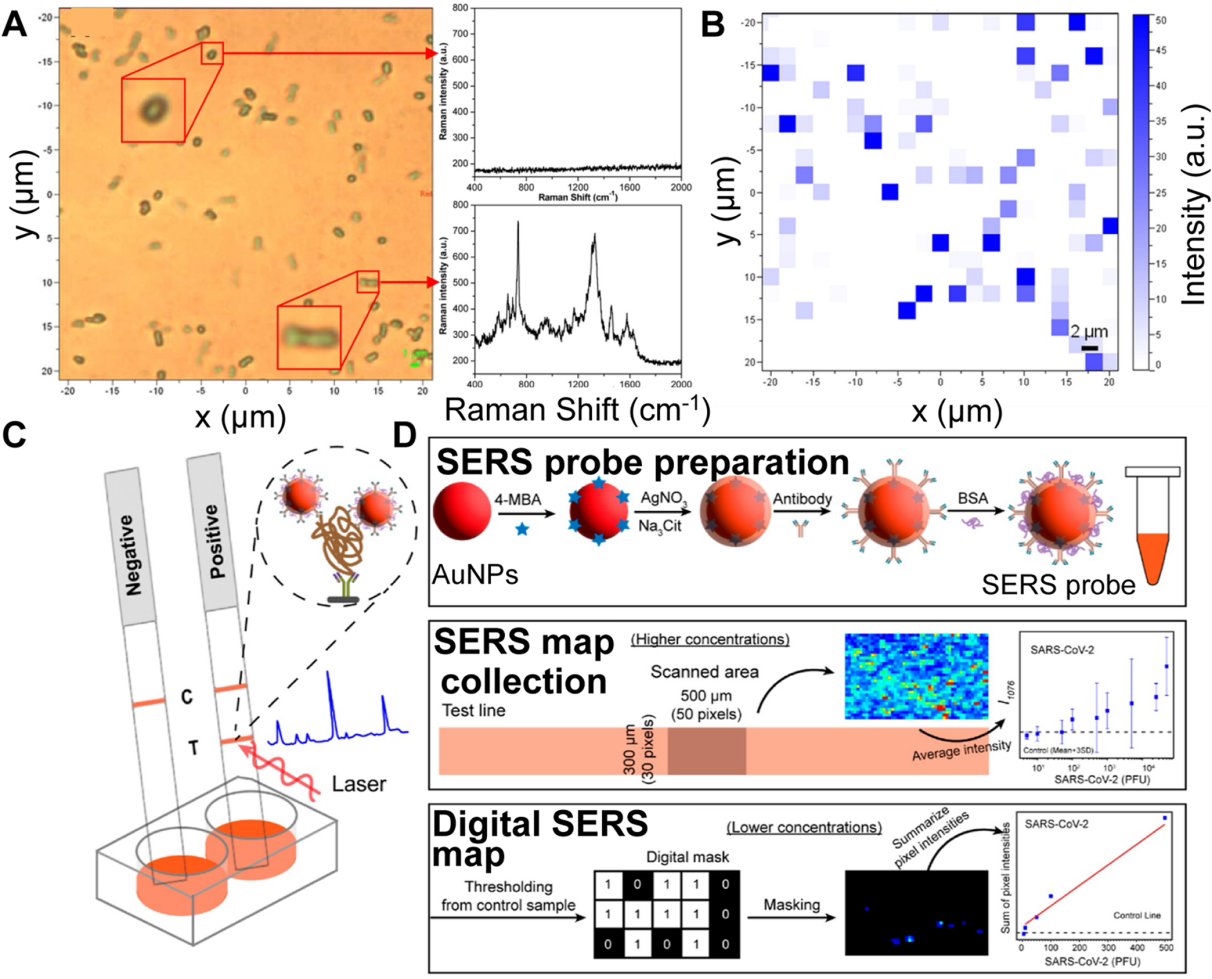
(A) Discrimination between single live and dead bacteria using the SERS spectrum of a single dead bacterium (top right) and a single live bacterium (bottom right). (B) Corresponding SERS image from (A). SERS mapping was constructed from the area of the strongest peak at 735 cm^–1^ (A and B are Reproduced from ref 120. Copyright 2015 American Chemical Society). (C) Illustrative overview of the SERS–Lateral Flow Test (LFT) dipstick for rapid SARS-CoV-2 detection. The SERS–LFT assay setup involves sample mixing with an antibody-functionalized SERS probe and running buffer within a 96-well plate, followed by SERS spectra collection at the test line. (D) Process of SERS probe preparation (top), 2D SERS mapping for viral quantification (middle), and 2D digital SERS mapping for low viral concentration quantification (bottom) (C and D are Reproduced from ref 122. Copyright 2024 American Chemical Society).

While label-free SERS imaging can provide fingerprint information, indicator-assisted SERS provides strong and distinct SERS signals. Ko et al. used the Raman indicator MGITC on 3D Ag@Au core–shell nanopillar arrays, fabricated via argon plasma etching and metal deposition, to map and quantify without culturing and enrichment.^121^ SERS signals from nanotags, created by immunoassays between *Salmonella* antibodies and the bacteria, allowed accurate statistical analysis. Building on a similar Au–Ag core–shell nanoparticle probe, we developed a digital SERS enabled lateral flow test dipstick. Through image processing methods, the substrate achieved ultrasensitive, rapid quantification of SARS-CoV-2 virus in real-world environmental settings (Figure 4C,4D).^122^ He et al. developed 3,3′-diethylthiatricarbocyanine iodide DTTC-conjugated gold–silver nanoshells for real-time SERS imaging of multidrug-resistant bacteria, enabling sensitive, noninvasive tracking of residual bacteria during wound healing.^123^ Bae et al. employed aryl-alkyne-based Raman tags to observe dynamic interactions between vancomycin and biofilms, demonstrating the value of indicator-assisted SERS in monitoring the inhibitory effects of antibiotics on biofilm growth in vivo.^124^ Zhou et al. incorporated terminal deoxynucleotidyl transferase (TdT)-catalyzed DNA into nanoprobes to create tunable nanogaps, enhancing SERS hotspots for high-resolution imaging.^125^ These nanogap-enabled probes provided high specificity and sensitivity for O157:H7 detection, with SERS images that were consistent with the distribution of cells in optical images.

SERS images of pathogens can also be used to probe cellular behavior and their interactions with proximal organisms. For example, Polisetti et al. used SERS imaging to visualize the distribution of the rhizosphere bacterium *Pantoea* sp. YR343 on the roots of .^126^ The SERS images revealed that high-intensity regions in the *Arabidopsis* heat map aligned with low-intensity regions in the *Pantoea* heat map, indicating a nonuniform distribution of the bacteria on the *Arabidopsis* root. Garg et al. developed Au–SiO_2_–Au nanolaminated plasmonic crystals (NLPCs) to conduct in situ spatiotemporal label-free SERS measurements of biofilms during biofilm development and upon infection by bacteriophage Phi6.^127^ Spatiotemporal of different biomolecules observed in the SERS spectra from lysed bacterial cell components, such as amino acids, nucleic acids, and lipids, can reflect the virus-specific alteration of the metabolism of host bacteria. Extending this approach, some researchers have combined SERS imaging with other technologies. Olson et al. obtained a high spatial resolution image of both Gram-positive and Gram-negative bacteria through an ultrathin silver substrate combined with SERS and stochastic optical reconstruction microscopy (STORM).^128^ These SERS-STORM images of bacteria showed excellent agreement with scanning electron microscope images, exhibited high spatial resolution at <50 nm, and showed that it was possible to correlate spectral SERS content to different regions of the bacterial cells. By comparing the SERS signatures of different bacteria, they found that Gram-negative bacteria exhibited greater lipid signatures than Gram-positive species due to their phospholipid lipopolysaccharide outer membrane.

## 4 SERS Imaging of Reactive Species in Biological and Environmental Matrices

Reactive species are critical due to their instability and high reactivity, which allows them to interact with and significantly influence their surrounding environments. These interactions play a key role in numerous biological and environmental processes, where they can drive important reactions, regulate cellular functions, and affect the stability and behavior of complex systems. Therefore, understanding reactive species is essential for both biological and environmental matrices. This section focuses on the characterization and quantification of two crucial categories of reactive species: protons (i.e., pH) and reactive oxygen species (ROS) since they dictate many biological and environmental processes. pH is defined as pH = −log_10_ *a*_H^+^_ = −log­(γ_H^+^_ *m*_H^+^_), where *a*_*H* ^+^_ denotes proton activity, γ_H^+^_ represents the ionic strength dependent activity coefficient, and *m*_*H* ^+^_ represents proton concentration. pH plays a pivotal role in chemical and biological processes in many biological and environmental systems. ROS typically includes singlet oxygen, superoxide radical, hydroxyl radical, and hydrogen peroxide.^129,130^ In biological matrices, hypochlorite ion, nitric oxide, and reactive nitrogen species are sometimes also considered ROS.^131^ Further, in environmental matrices, carbonate radicals are sometime regarded as ROS.^132^ SERS has enabled advanced detection and imaging of pH and ROS, typically through indicator-assisted methods. As shown in Figure 5A, the design and optimization of pH and ROS involves specific reactions between nanosensors and the target molecules (H^+^ or specific ROS) and must ensure compatibility within the target system. Recently, new SERS probes have been developed, one example being a SERS-borrowing-strategy-based nanoprobe (Au@Pt core–shell nanoparticles), which enables the simultaneous and direct identification of different mitochondrial ROS based on their distinct Raman fingerprints.^133,134^

**Figure 5:**
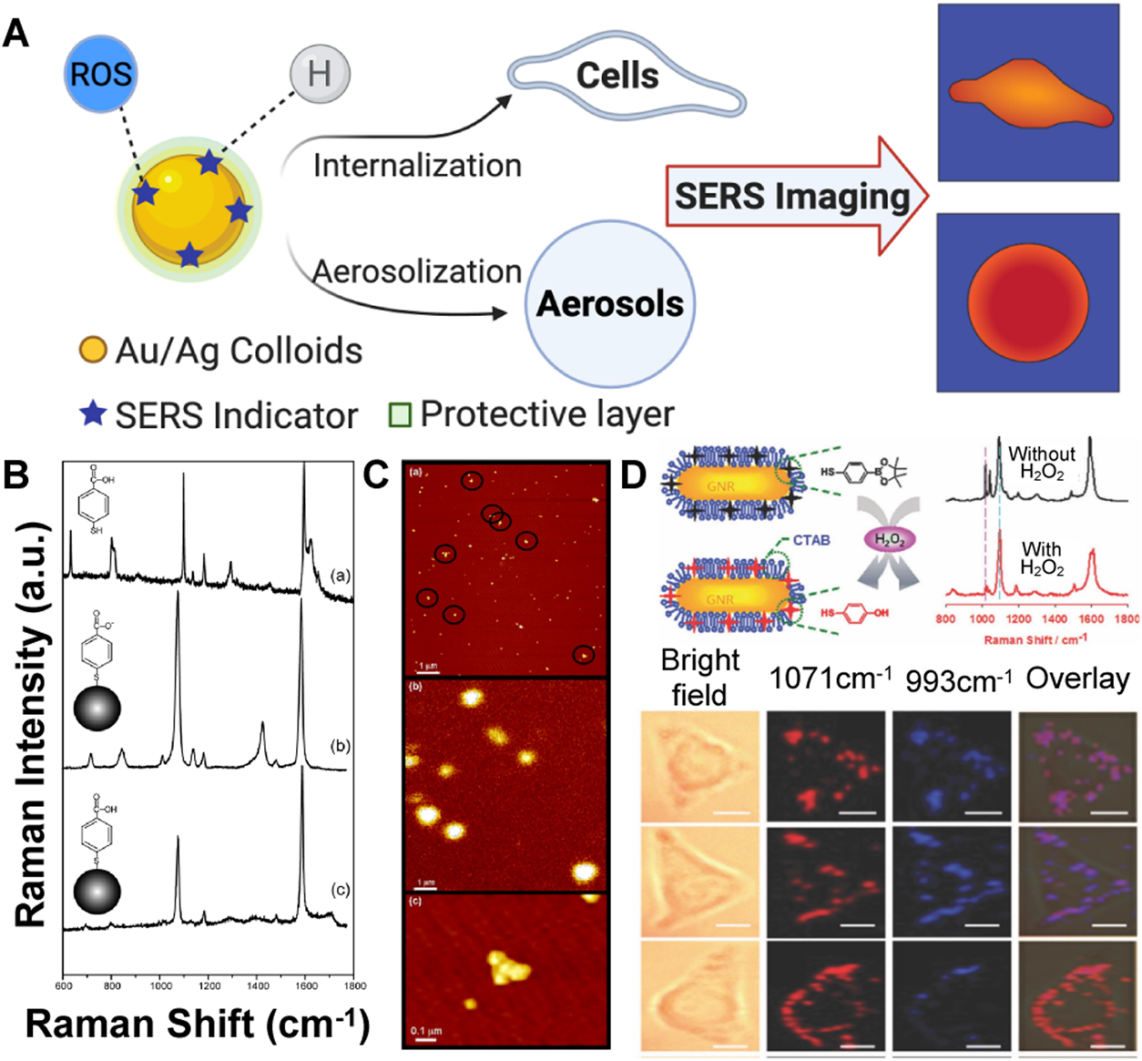
(A) Schematic of the design of SERS nanosensors for pH and ROS detection; (B) the Raman spectra of solid 4-mercapto benzoic acid (4-MBA), and the SERS spectra of pH nanoprobe (4-MBA-silver nanoparticles) at pH = 12.3 and pH = 5.0; C. AFM and confocal optical images of 4-MBA- functionalized nanoparticles immobilized on a salinized glass coverslip. (B and C Reproduced from ref 135. Copyright 2004 American Chemical Society); (D) SERS nanoprobe (4-mercapto-phenylboronic ester (MPBE) functionalized Gold nanorod (GNR)) for H_2_O_2_ imaging in living cells or cancer tissues. Bottom images are confocal ratiometric SERS images with 10 mm scale bar of live HeLa cells. Images displayed in pseudocolor represent the ratio of SERS intensities collected in 1071 cm^–1^ (red) and 993 cm^–1^ (blue), respectively (Reproduced from ref 155. Copyright 2016 Royal Society of Chemistry).

### 4.1 SERS Imaging for pH

pH nanoprobes are typically made from Au or Ag colloids with a pH-sensitive indicator molecule selected for its p*K*_a_ to match the desired pH range. Specific binding between protons and indicators causes changes in the SERS spectrum thus indicating the pH in the vicinity of nanoprobe. Talley et al. first applied AgNPs functionalized with 4-mercaptobenzoic acid (4-MBA) for intracellular pH measurement in 2004 (Figure 5B,5C).^135^ Later, Kneipp et al. substituted AgNPs with AuNPs due to their increased biocompatibility and stability.^136^ They reported a pH response range of 2–8 for 4-MBA-functionalized AuNPs, which then became widely used.^137^ Amine-based indicators such as 4-ATP and 2-ATP, with higher p*K*_a_ values, extended the effective range to ∼4–10.^29,34,138,139^ Jensen et al. demonstrated that both amine and pyridine groups (e.g., in 4-mercaptopyridine) could serve as pH indicators.^30^ Kong et al. developed a gold-coated nanoroughened planar substrate with arene chromium tricarbonyl linked aminothiophenol (Cr­(CO)_3_-ATP) being the pH indicator.^140^ The substrate-based sensor avoids nanoparticle aggregation; however, the immobilized nanoprobe cannot be used for intracellular study.

Nanoprobe stability is crucial for reliable SERS pH sensing in biological matrices, since nanoparticle aggregation and biomolecule adsorption can affect signal quality. Protective layers are often applied to enhance stability and prevent aggregation. Chen et al. developed a hybrid structure with core–shell Au@AgNPs and multiwalled carbon nanotubes (MWCNT).^139^ This hybrid pH nanoprobe increased the number of SERS hotspots and expanded the pH measurement range while maintaining stability. Wang et al. coated the pH sensitive core with BSA as a protective layer to improve biocompatibility and stability.^141^ A similar approach was adopted by Zheng and colleagues for AuNP-4-Mpy-BSA nanoprobes for pH imaging.^22^ However, the cell internalization efficiency of the BSA-coasted nanoprobes remained low, and the nanoprobe was trapped in lysosomes thus resulting in heterogeneous distribution of the nanoprobe. To address this, the Zhang group conjugated BSA-coated nanoprobes with a cell-penetrating peptide (cysteine-terminated Tat peptide), allowing long-term pH studies in cells.^14^ The cationic Tat peptide can penetrate the negatively charged plasma membrane of human cervical cancer cells directly without the participation of endocytosis, which greatly enhance the uptake of pH probes without sacrificing the pH response. Other strategies, including ultrasound-mediated method and electroporation technique, have also been reported to improve nanoprobe internalization efficiency.^142^ The stability and sensitivity of the pH nanoprobes can be affected by the presence of certain ions in the surrounding environment.^143^ Guo et al. found that interference from halide ions on 4-Mpy nanoprobes was minimized by prefunctionalizing with bromide ions before applying a polyethylene glycol layer, electrostatically stabilizing the protonated 4-Mpy.^144^

Intracellular studies demonstrates that SERS imaging is a valuable tool for studying microenvironments. In environmental matrices, the pH of microenvironments such as individual atmospheric aerosols can be quantified.^145,146^ Our group developed a pH-sensitive AuNP-based nanoprobe with 4-MBA and polyethylene glycol, achieving 3D pH mapping in phosphate-buffered microdroplets and revealing a pH gradient from a centroid pH of ∼11, decreasing toward the surface (bulk pH ∼ 7.4).^147^ We applied the pH nanoprobe to ammonium-based aerosol droplets, observing a homogeneous pH increase with ammonium concentration, which reveals the role of ammonium in aerosol pH.^16^ We also employed flash-freezing for pH measurement in environmental water droplets,^17^ advancing knowledge of ion distribution. While SERS offers sufficient spatial resolution for micrometer-sized systems, its application to submicron aerosol droplets remains limited.^146,148^ Recent studies have advanced SERS spatial resolution for finer aerosols; for example, Craig et al. resolved chemical variations within 0.5 μm in 1 μm aerosol particles using a 50 nm Ag nanosphere-coated quartz substrate, underscoring the potential of SERS for single-particle imaging of fine-mode aerosols.^148^ However, debate persists over aerosol droplet pH distribution: Li et al. observed homogeneity in phosphate and ammonium sulfate droplets using optical tweezers and Raman imaging,^149^ while stimulated Raman spectroscopy (SRS) revealed a heterogeneous pH distribution at the air–water interface due to HSO_4_^–^.^150^ These contrasting observations highlight the need for further research to reconcile findings and ensure consistency across SERS and related Raman imaging techniques.

### 4.2 SERS Imaging for Reactive Oxygen Species (ROS)

ROS play essential roles in cellular activities, including metabolism, signaling, gene expression, and immune responses, and serve as indicators of oxidative stress.^151,152^ Ideal SERS probes for ROS detection incorporate indicators that specifically react with target ROS to enable precise detection, identification, localization, and quantification.^131^ SERS-based ROS sensing is typically reliant on Au/AgNP colloids with functional groups that react with ROS, though development is challenging due to ROS’s high reactivity and short lifetimes.^131^

Chen et al. detected H_2_O_2_ and the H_2_O_2_ scavenging activity of tannic acid and L-Malic acid by applying SiO_2_/AuNP nanoshells as nanoprobes.^153^ This study utilized the reductive ability of H_2_O_2_ on Au^3+^ and found a correlation between H_2_O_2_ concentration and SERS intensity upon the formation of Au^0^ on the SiO_2_ surface. In living cells, boronate-based ratiometric nanoprobe, such as 3-mercaptophenylboronic acid (3-MPBA),^154^ 4-mercaptophenylboronic ester (MPBE),^155^ and 4-carboxyphenylboronic acid (4-CA),^156^ have been applied as H_2_O_2_ indicator, since boronated molecules can specifically react with hydrogen peroxide. Upon reaction with H_2_O_2_, the correlation between the H_2_O_2_ concentration and the peak-ratio between the ROS-responsive and ROS-nonresponsive peaks can be used to quantify H_2_O_2_. In the study with MPBE, SERS imaging of H_2_O_2_ was overlaid with a dark-field microscopic (DFM) image to generate a 2-D profile in live HeLa cells (Figure 5D).^155^ Z-scan SERS imaging further assessed H_2_O_2_ penetration depth in rat cervical tumor tissue slices, with high H_2_O_2_ response correlating to the HeLa cell edge. Dong et al. developed another method detecting H_2_O_2_ and glucose by quenching the SERS signal through a specific reaction between ROS and methylene blue (MB) absorbed onto starch-coated Au nanoshells (GNSs), presenting a novel approach to ROS detection.^157^

Other types of ROS have been detected using nanoprobes with appropriately designed indicators. Superoxide anion radicals were detected due to their ability to modify oxidized cytochrome c (Cyt c) on AuNPs. An intermediate molecule, 3-mercaptopropionic acid (3-MPA), was used to bridge the Cyt c and AuNPs.^158^ Molecules containing phenol groups, such as 2-mercapto-3methoxy-phenol and 4-mercaptophenol,^159,160^ have been applied for hypochlorite sensing. Cao et al. achieved carbon monoxide (CO) sensing by modifying the AuNP surface with synthesized palladacycles.^161^ The CO nanoprobe was used for the observation of CO-dependent carbonylation.

Changing from biological matrices into environmentally relevant systems, spontaneous production of OH radicals was observed using a phthalhydrazide-functionalized AgNP probe in water microdroplets.^162^ However, the application of SERS imaging for mapping ROS in environmental matrices remains largely underexplored. Given the critical role of ROS in various environmental processes, there is significant potential for expanding the use of SERS imaging to study ROS-driven reactions and mechanisms in environmental systems.

## 5 Applications of SERS Imaging in Environmental Sensing

Building on the advancements in SERS imaging for biological and reactive species analysis, the application of SERS in environmental research faces unique challenges due to the complexity of environmental systems. While several review articles have addressed the application of SERS for analyzing environmental pollutants, few have explored the SERS imaging potential for environmental sensing.^163–165^ This gap can be attributed to two main reasons: (1) in general, environmental monitoring studies have focused on whether or not a pollutant is present and how high its concentration is, which usually can be achieved without imaging. However, the spatial distribution of environmental pollutants can have significant implications for environmental fate and safety; (2) despite the widespread use of SERS imaging in biological applications, it remains relatively underexplored in the environmental field. Despite these challenges, SERS imaging has been successfully applied to detect and map three major types of environmental contaminants: pesticides, noble metal nanoparticles, and heavy metal ions.

### 5.1 Pesticides

Pesticides are commonly used agrochemicals due to their capacity to reduce crop losses, improve overall food productivity, and increase agricultural profit. However, during or after application, pesticides may result in environmental contamination, such as the residues on crops and vegetables. To evaluate pesticide risk to environmental safety and public health, it is important to evaluate their uptake, translocation, and degradation within agricultural products.

SERS imaging has become an effective tool for tracking pesticide penetration in plants. For example, SERS depth mapping was used to monitor two pesticides in spinach leaves: the systemic pesticide thiabendazole, which penetrates plant tissues, and the nonsystemic pesticide ferbam, which remains on the surface.^23^ Depth mapping revealed that thiabendazole signals were detectable within leaf cross sections, while ferbam was only observed on the surface, confirming their respective properties (Figure 6A).^23^ Similar penetration behaviors were found in basil leaves, with differences in speed and depth between live and harvested samples.^166^ Extending this approach, researchers tracked four pesticides (thiabendazole, acetamiprid, ferbam, and phosmet) in apples, grapes, and spinach, finding that nonsystemic pesticides penetrated produce, but at less rapid and shallower levels compared to systemic pesticides.^167^ Additionally, SERS surface and depth imaging evaluated different washing methods for pesticide removal from apple surfaces, showing that NaHCO_3_ solution was most effective, though internalized pesticides remained unaffected.^168^ Since pesticides such as thiabendazole and ferbam can adsorb onto AuNPs via strong Au–S bonds, AuNPs may serve as carriers, potentially altering penetration patterns within plant tissues.^23,169^ Based on this, an AuNPs (50 nm) mirror was developed to map pesticides on tomato leaves, fruits, and mouse skin via SERS imaging (Figure 6B).^170^

**Figure 6:**
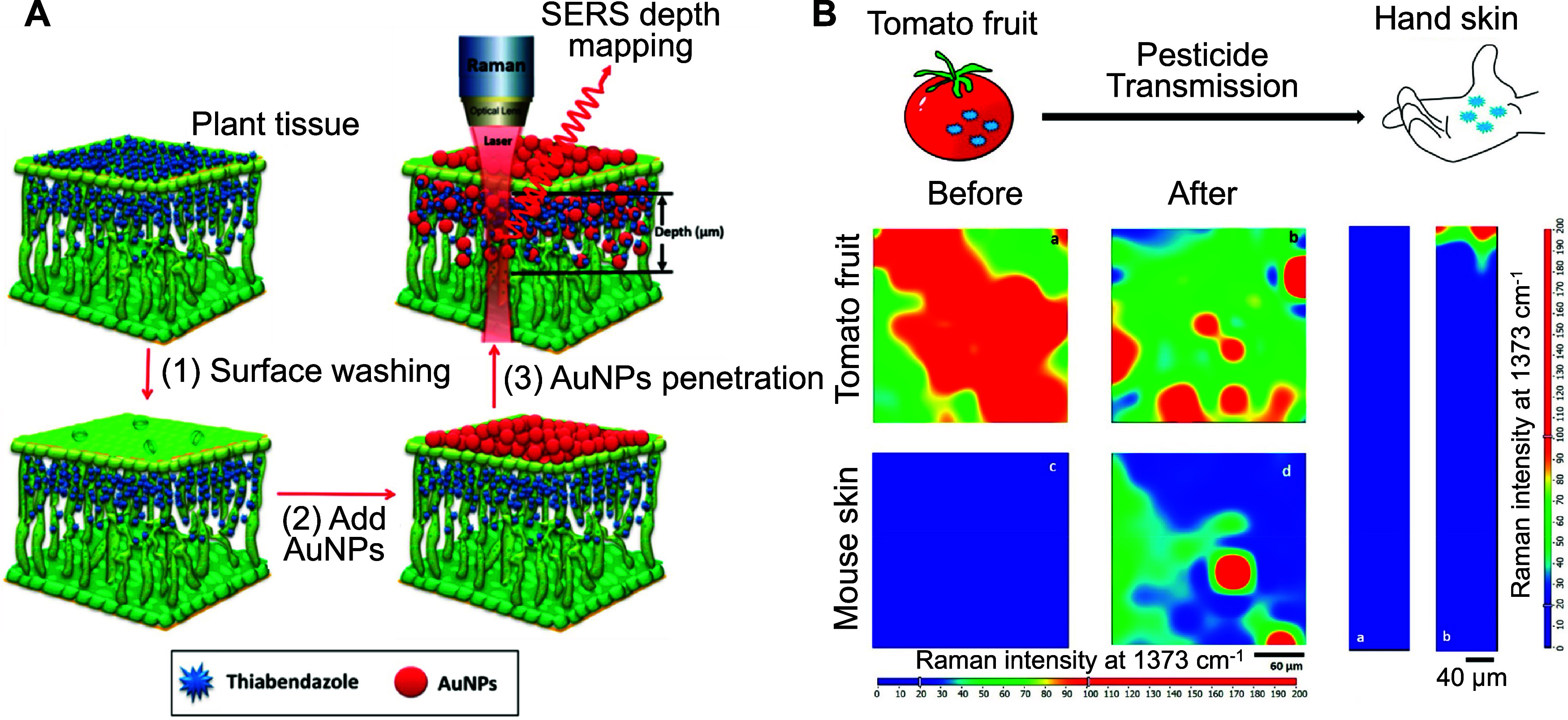
(A) Schematic illustration of thiabendazole penetration using SERS mapping technique. (Reproduced from ref 23. Copyright 2016 American Chemical Society). (B) Schematic presentation of pesticide transmission from tomato fruit to the palm of the mouse hand, and SERS surface mapping images of pesticides on tomato fruit before and after transmission, and on the mouse hand before and after transmission. (Reproduced from ref 170. Copyright 2019 American Chemical Society).

### 5.2 Nanoparticles

Nanoparticles (NPs), including heavy metal NPs and nanoplastics are considered potential contaminants, but their characterization and quantification are challenging due to their small size and matrix complexity. SERS is an innovative tool for NP imaging, especially for noble metal NPs due to their significant Raman enhancements. SERS imaging of NPs in complex materials (e.g., plant matrices) can be indicator-free or indicator-assisted. Indicator-free methods are ideal for studying NP chemical transformations, surface chemistry, or interactions within the matrix, while indicator-assisted approaches are preferred for sensitive identification and quantification of NPs.

In indicator-free SERS imaging, the enhanced Raman signals of molecules surrounding NPs indicate the presence of NPs, and deliver important information on the interactions between NPs and localized environments. Zhang et al. used SERS imaging for in situ qualitative detection and characterization of AuNPs on and in spinach leaves,^171^ identifying AuNPs by Raman signals from leaf biomolecules like chlorophylls and carotenoids. SERS mapping revealed AuNPs distributed on the leaf surface and penetrating leaf tissues, where they interacted with plant biomolecules. In single-cell applications, Kawata et al. demonstrated in vivo real-time SERS monitoring of AuNP transport within single living algal cells.^172,173^ The Brunner group used 3-D SERS maps to study AuNP uptake by the diatom (), observing size-dependent localization: AuNPs smaller than 50 nm remained near the cell wall, while larger particles penetrated the diatom.^174^ SERS signals from pigments such as chlorophyll a, fucoxanthin, and β-carotene helped map the chloroplast locations, aligning closely with cell shape and allowing detailed cell dimension analysis. SERS imaging can also track the in situ formation of AuNPs within cells. Our group applied SERS to monitor AuNP biosynthesis in algae exposed to HAuCl_4_ under various pH conditions.^24^ Two- and three-dimensional SERS maps revealed the location and local molecular environment of the synthesized AuNPs, with surface-associated biomolecules identified as glutathione, chlorophyll a, hydroxyquinoline, NAD, and reductase enzymes. This method was faster and less invasive than traditional electron microscopy, with minimal sample preparation required. Similarly, Pytlik et al. used in vivo SERS imaging to study AuNP formation in ,^175^ finding that AuNPs formed primarily inside the diatom, surrounded by biomolecules such as hydroxyurea and retinol, distinct from those in green algae.

Indicator-assisted SERS imaging is required when the original ligands on NPs are not Raman active. To image NPs in real environments, selection of indicators with strong binding ability to the NPs is crucial to minimize interference and improve detection sensitivity. The basic rationale for indicator selection is that the indicator should have (1) high and distinct Raman peaks to achieve high sensitivity; and (2) strong binding ability with nanosubstrates to enhance specific adsorption and minimize interference.^176^ Indicator-assisted SERS imaging has been used to track the internalization and localization of heavy metal nanoparticles in living animals. For instance, Zavaleta et al.^177^ labeled Au/silica core/shell structure with 10 different types of Raman-active indicators in between the core and shell. They achieved multiplexed SERS imaging of differently labeled NPs simultaneously in the skin and liver of a live mouse. This study provides versatile nanocarriers that offer multiple loading/delivery options with sensitive SERS imaging.

SERS imaging can also be used to track nanoplastics. A recent study examined the intestinal barrier translocation of nanoplastic pollutants in Daphnia through confocal SERS imaging. The nanoplastics were made of Raman-indicator-labeled AuNPs as the core and polystyrene as the shell. This new approach enables in situ tracking of the nanoplastic translocation across the gut in the organism.^178^ In another work, the authors characterized the penetration of organic pollutants into microplastics using SERS imaging. By employing AuNPs as signal enhancers, the study visualized the spatial and temporal dynamics of pollutant penetration into microplastic matrices. The methodology improves our understanding of contaminant sorption processes and their distributions in the environment.^179^

### 5.3 Heavy Metal Ions

Heavy metal ions exist in many natural and man-made environments, with some essential for life, but toxic at high concentrations, and some poisonous even at low concentrations. To track the environmental fate and behavior of heavy metal ions, it is necessary to monitor their transport and transformation in biological cells. However, different from noble metal nanoparticles, metal ions are more challenging to detect due to the lack of significant Raman enhancement.

Some heavy metal ions, such as Cr­(VI) and Cr­(III), have apparent SERS bands. Using Cr­(VI) functionalized AuNPs, Ravindranath et al. created an intracellular sensing platform that mapped sites of reduced Cr­(III) and unreacted Cr­(VI).^180^ However, detection was limited by the localization of internalized AuNPs. To address this, they developed a platform combining confocal Raman imaging with in situ biosynthesized gold nanoislands, which acted as SERS nanostructures.^25^ This approach allowed for the identification of both Cr­(VI) and Cr­(III) within a single bacterial cell by enhancing characteristic peaks through the nanoislands. The major breakthrough of this work was its ability to differentiate between Cr­(VI) and Cr­(III) and to map Cr­(VI) reduction to Cr­(III) at single-cell resolution.

Indicator-assisted SERS methods have been developed for heavy metal ions that do not produce distinguishable SERS fingerprints, though few studies currently focus on their application in heavy metal ion imaging.^37,38,181,182^ Heavy metal ions can be quantified based on the changes in indicator signal patterns or intensities upon interaction with heavy metal ions.^181,182^ For example, 4-Mercaptopyridine-labeled AuNPs (4-MPy-AuNPs) enabled the detection of Hg^2+^ and CH_3_Hg^+^ due to the distinct SERS peaks of 4-Mpy, 4-Mpy-Hg^2+^ and 4-Mpy-(Hg-CH_3_)^+^.^182^ Another strategy uses peak intensity changes,^37,38^ as seen with Cd­(II), where the addition of Cd­(II) causes AuNP aggregation, creating more SERS hotspots and enhancing alizarin dye signals.^38^ Selective coordination of Cd­(II) with surface molecules like 3-mercaptopropionic acid on AuNPs promotes aggregation and detection specificity. Future research may explore these strategies in SERS imaging to study heavy metal ion uptake, distribution, and transformation in living cells or microenvironments.

## 6 Conclusions and Future Perspectives

SERS imaging has emerged as a powerful analytical tool, providing high-sensitivity, photostable biomolecular fingerprinting with precise localization, ideal for both biological and environmental applications. The current state of the art in SERS imaging is reflected in the following aspects: (1) Significant advances in SERS nanoprobes, optimized for pH stability, low toxicity, and effective NIR Raman reporters, demonstrates great potential for multiplexed imaging and targeted biomarker detection, including human cells. Additionally, target-specific functionalization enables SERS imaging of reactive species in biological and environmental microenvironments (e.g., proton/pH and ROS) with high sensitivity, selectivity and stability. (2) Integration with complementary imaging techniques (e.g., PA, MRI) along with the development of flexible hand-held scanners, further expands its applicability. (3) The multifunctionality of SERS enables applications in drug delivery and theranostics, which promises advancements in clinical diagnostics. (4) In environmental sensing, SERS imaging has proven effective in contaminant trapping and mapping (e.g., pesticides, heavy metal nanoparticles, and ions), providing valuable insights into their transport and transformation in complex environmental systems.

The applications of SERS imaging in biological and environmental systems offer several key advantages: (1) in situ and real-time detection, (2) high sensitivity to a wide range of analytes, (3) fluorescence-free nondestructive analysis, and (4) resolve spatial distribution and temporal evolution. However, some limitations remain and require further optimization:

(1) Dependence on nanoprobes. Colloid-based SERS imaging relies heavily on the presence of Au nanoprobes, meaning that without nanoprobes, analytes cannot be detected. Consequently, SERS maps reflect areas where nanoprobes and analytes, such as pesticides, coexist rather than showing the analyte distribution alone.
(2) Potential alteration of analyte. The introduction of nanoprobes can potentially alter analyte distribution, which must be considered in SERS imaging studies. Colloidal Au nanoprobes may form arbitrary aggregates upon introduction, leading to uneven distribution and heterogeneous penetration in plant tissues, which can bias SERS imaging results. Further, SERS spectra may change upon the addition of nanoparticles (NPs), due to condition-dependent NP-cell interactions and the ROS generation caused by the NP dose.^183^
(3) Selectivity in complex systems. Achieving selectivity in complex systems is challenging. For example, pH-sensitive nanoprobes like 4-MBA may coordinate with metal ions, while ROS sensors relying on redox reactions may be confounded by other ROS with similar redox potentials.
(4) Challenges in quantitative analysis. Quantitative SERS imaging is underexplored, as calibration standards created in solution in lab may not be directly applicable to real imaging environments. Improved quantification strategies are needed to enhance the accuracy of SERS imaging.

Specifically, SERS imaging for biological analytes also faces several challenges that need to be addressed for further development:

(1) Challenges in label-free SERS imaging. Label-free SERS imaging generates spectra that capture signals from all present biomolecules, which can complicate interpretation in the complex and dynamic intracellular environment of living cells. This may lead to missed or distorted findings. Advanced algorithms, such as machine learning, could be applied to extract meaningful patterns from spectral data and improve the reliability of label-free SERS imaging in such intricate settings.^184^ AI-assisted spectral analysis and modeling have been reported to monitor real-time interactions between the SARS-CoV-2 and human ACE2 based on their IR spectra.^185^ However, how it can be used for real-time SERS spectral analysis in biological samples is unexplored. In the future, it is worthwhile to develop SERS-specific machine learning models to facilitate the SERS imaging data interpretation. For example, machine learning methods can be used to identify different biomarkers and characterize their distributions based on their SERS spectra and microscopic maps in biological samples.
(2) Pathogen Detection Sensitivity. The relatively large size of many pathogens compared to the SERS hot spots generated by substrates or nanoprobes can result in reduced sensitivity and inconsistent results; therefore, the reproducibility needs to be improved for large target analytes. Additionally, certain biomolecules of interest, such as proteins or lipids, have low Raman cross sections, further limiting sensitivity. To address these challenges, strategies to enhance SERS signals, such as developing adaptable, 3D-structured SERS substrates, may improve compatibility with pathogen dimensions, thereby enhancing detection sensitivity and reproducibility.
(3) Capturing rapid biomolecular changes. The highly dynamic nature of living cells, where biomolecules can redistribute or transform within seconds due to various physiological activities, poses a challenge for SERS platforms in capturing rapid, spatial, and temporal changes accurately. Ensuring that SERS imaging can reflect these variations in real-time is critical for its effectiveness in cellular studies. Moreover, the SERS signals of biological samples have been observed vary over time due to the changes in the surrounding environment.^186,187^ Therefore, maintaining a stable environmental condition and implementing rapid scanning are essential for ensuring data reliability, particularly in large areas or 3D imaging applications.
(4) Nanoparticle accumulation and elimination. Ensuring normal cell viability during SERS imaging is the trend for single living cell studies which reveals the true nature of life. However, current development for SERS probes functionalization (e.g., new cell-penetrating peptides) focus mostly on the internalization efficiency, little attention has been paid to the toxicity and elimination of SERS imaging agents within organisms. Even though many SERS-active nanoparticles are considered low in toxicity, their potential accumulation within living cells or tissues raises concerns about long-term effects. Developing strategies for the safe elimination of accumulated nanoparticles should be a focus in future research to ensure biocompatibility. Other challenges for SERS imaging in intracellular sensing includes the spectra change caused by the addition of nanoparticles (NPs), condition-dependent NP-cell interactions and the ROS generation caused by the NP dose.^183^

For environmental applications, SERS imaging remains in its early stages but holds significant potential to improve in situ, real-time investigation of contaminants. As environmental systems are open and dynamic, SERS imaging must overcome challenges like complex sample matrices and tracking the spatial and temporal evolution of pollutants. Addressing these issues will expand the potential of SERS for more comprehensive environmental analysis. Additionally, future research should expand SERS imaging to include emerging contaminants, such as endocrine-disrupting chemicals (EDCs), pharmaceuticals and personal care products (PPCPs), brominated flame retardants, and microplastics. Through continued analytical advances, SERS imaging has potential to become a robust tool for tracking contaminants and assessing environmental impacts.
